# mRNA 3′end processing: A tale of the tail reaches the clinic

**DOI:** 10.1002/emmm.201303300

**Published:** 2013-10-09

**Authors:** Ina Hollerer, Kerstin Grund, Matthias W Hentze, Andreas E Kulozik

**Affiliations:** 1 Department of Pediatric Oncology, Hematology and Immunology, University of HeidelbergHeidelberg, Germany; 2 Molecular Medicine Partnership Unit (MMPU)Heidelberg, Germany; 3 European Molecular Biology Laboratory Heidelberg (EMBL)Heidelberg, Germany

**Keywords:** clinical importance of 3′ end mRNA processing, control of polyadenylation, post-transcriptional gene regulation, regulation of 3′ end mRNA processing

## Abstract

Recent advances reveal mRNA 3′end processing as a highly regulated process that fine-tunes posttranscriptional gene expression. This process can affect the site and/or the efficiency of 3′end processing, controlling the quality and the quantity of substrate mRNAs. The regulation of 3′end processing plays a central role in fundamental physiology such as blood coagulation and innate immunity. In addition, errors in mRNA 3′end processing have been associated with a broad spectrum of human diseases, including cancer. We summarize and discuss the paradigmatic shift in the understanding of 3′end processing as a mechanism of posttranscriptional gene regulation that has reached clinical medicine.

## Introduction

Until recently, mRNA 3′end processing was viewed as a constitutive step of RNA biogenesis. Current advances have shown that the mechanism is regulated via a network of *cis*-acting RNA sequence elements and *trans*-acting proteins, contributing to the qualitative and quantitative adjustment of gene expression (Millevoi & Vagner, 2010). Reports about the impact of regulated mRNA polyadenylation and cleavage on physiological as well as pathological pathways increase steadily. These days, the underlying molecular mechanisms are the subject of intensive research. In this review, we provide the background on the recent progress in the field, leading to a better understanding of the complex process of mRNA 3′end processing; more detailed reviews on the mechanism of constitutive 3′end processing and alternative polyadenylation site (PAS) choice have recently been published (Elkon *et al*, 2013; Tian & Manley, 2013). We will especially highlight quantitative regulation of 3′end processing and its role in human pathophysiology.

## Molecular mechanisms of mRNA 3′end processing

### Constitutive 3′end processing

**Figure 1:**
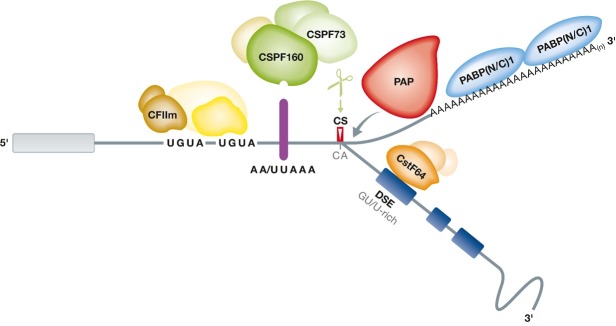
Endonucleolytic cleavage of pre-mRNAs and their subsequent polyadenylation requires the four multisubunit complexes CPSF, CstF, cleavage factor I (CFIm) and cleavage factor II (CFIIm) as well as the single-subunit polyadenylation-polymerase PAP. At the PAS CPSF and CstF bind to the central hexameric sequence (AA/UUAAA) and to the GU/U-rich DSE, respectively, as the first step in mRNA 3′end processing. The other protein complexes assemble at specific RNA sequence elements both up- and down-stream of the PAS, including several UGUA-repeats, thereby ensuring efficient cleavage and polyadenylation of an mRNA. The pre-mRNA is cleaved at the CS by the endonuclease CSPF73 before the nuclear poly(A) polymerase adds ∼200 As to the 3′end. This poly(A) tail stabilizes the processed mRNA for nuclear export upon binding of the nuclear poly(A) binding protein (PABPN1) which is subsequently exchanged for its cytoplasmic counterpart PABPC which promotes translation and RNA stability.

As the first step in the maturation of eucaryotic mRNAs, transcripts are synthesized by RNA polymerase II (PolII), capped, spliced and polyadenylated. Cleavage and polyadenylation of cellular pre-mRNAs at the 3′end is accomplished by a fine-tuned mechanism involving a number of RNA-binding proteins (RBPs) and regulatory *cis*-acting RNA sequence elements (Fig 1). In addition to the core machinery required for mRNA 3′end processing, auxiliary regulatory protein factors, miRNAs and supporting RNA sequence elements contained in the 3′-untranslated region (3′UTR) ensure efficient processing of a target mRNA (Matoulkova *et al*, 2012; Millevoi & Vagner, 2010). The processing steps of tRNA and replication-dependent histone mRNAs differ from the mechanism described here and are extensively covered elsewhere (Maraia & Lamichhane, 2011; Yang *et al*, 2013).

#### *cis*-acting RNA elements required for 3′end processing

In an mRNA's 3′UTR, the arrangement of *cis*-acting sequence elements determine the efficiency of a poly(A) (polyadenylation) site.

The most prominent among these *cis*-acting elements is a hexameric sequence motif that was first described by Proudfoot and Brownlee (1976). In ∼70% of human RNAs, the hexamer consists of the nucleotide sequence AAUAAA or AUUAAA. The remaining ∼30% of RNAs contain other sequences, such as UAUAAA, AACAAA, or ACUAAA (MacDonald & Redondo, 2002). The “strength” of a specific PAS is determined by surrounding auxiliary RNA elements occurring both down- and upstream of the hexamer, which serve as binding platforms for core and auxiliary 3′end processing factors (Millevoi & Vagner, 2010). The PAS not only affects mRNA 3′end processing but also plays an important role in other steps of the gene expression pathway, such as coupling 3′end processing to transcription termination (Proudfoot *et al*, 2002).

A less well-conserved auxiliary *cis*-acting element is the U/GU-rich downstream sequence element (DSE), which is located 30 to 45 nt 3′ of the hexameric sequence motif (Gil & Proudfoot, 1987). As a first step in mRNA 3′end processing, the cleavage and polyadenylation specificity factor (CPSF) and the cleavage stimulation factor (CstF) bind to the hexamer and the DSE, respectively, and stimulate polyadenylation (Keller *et al*, 1991; MacDonald *et al*, 1994; Proudfoot, 2011). The relative positions of the hexamer and the DSE define the site of cleavage of a pre-mRNA, which typically occurs within a 13-nucleotide window between these two elements (Chen *et al*, 1995). Most pre-mRNAs are cleaved 3′ of an adenosine residue and CA is defined as the optimal cleavage site (CS), although the nucleotide sequence of the exact CS is not highly conserved (Sheets *et al*, 1990).

Additional auxiliary *cis*-acting elements located both downstream and upstream of the central hexamer have been reported to be involved in mRNA 3′end processing, including U-, GU- and AU-rich sequences (Tian & Graber, 2012).

#### Protein factors involved in constitutive 3′end processing

Efficient mRNA 3′end formation is ensured by a network of interacting proteins. Although the three-dimensional structure of this machinery is still unknown, its ∼20 core proteins have been identified by biochemical analyses. This protein complex not only ensures efficient mRNA cleavage and polyadenylation, but also links 3′end formation to other steps of mRNA biosynthesis and processing (Shi *et al*, 2009b). Endonucleolytic cleavage of pre-mRNAs and their subsequent polyadenylation requires the four multisubunit complexes CPSF, CstF, CFIm and CFIIm, referred to as the core 3′end processing factors, and the single-subunit nuclear poly(A)-polymerase PAP plus the nuclear poly(A)-binding protein (PABPN) (Millevoi & Vagner, 2010). Additional factors stimulating the polyadenylation of a nascent mRNA comprise RBBP6 and the serine/threonine phosphatase PP1α/β (Shi *et al*, 2009a).

### Mechanisms of regulated 3′end processing

**Figure 2:**
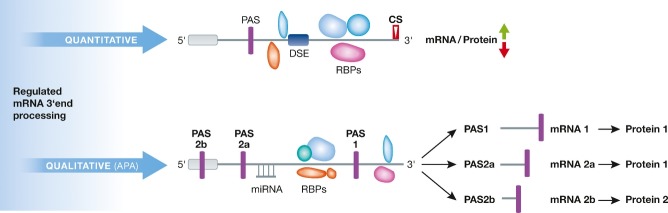
In a quantitative manner, the regulation of 3′end processing stimulates or inhibits the gene expression via the formation of specific mRNPs. Qualitatively, mRNAs containing more than one polyadenylation signal (PAS) can be subjected to APA. In case both PAS are present in the 3′UTR (PAS1 and PAS 2a), APA results in the expression of mRNA isoforms that encode the same protein (protein 1) but differ in the length of their 3′UTR (mRNA 1, mRNA 2a), including or excluding regulatory elements such as miRNA or RBP binding sites. If one poly(A) signal is contained within the coding region (PAS 2b), APA produces mRNA isoforms with distinct C-terminal coding regions (mRNA 2a and mRNA 2b), which leads to the expression of different protein isoforms (protein 1 and protein 2).

Regulated 3′end processing has attracted attention because gene expression can be adjusted in a quantitative manner by tuning the amount of polyadenylated mRNA and protein produced (Millevoi *et al*, 2009; Wahle & Ruegsegger, 1999). In addition, ∼70% of human pre-mRNAs and ∼66% of long noncoding RNAs contain more than one functional polyadenylation signal and are candidates for alternative polyadenylation (APA) (Tian & Manley, 2013) (Fig 2). Depending on the location of the alternative PAS relative to the exon/intron structure of the pre-mRNA, APA either triggers the production of mRNA isoforms differing in the length of their 3′UTRs or varying in their C-terminal coding region. The former frequently entails a regulation of gene expression via the exclusion or inclusion of regulatory elements such as miRNA- and RBPs-binding sites, whereas the latter contributes to the diversity of the proteome. Current research focuses on the mechanisms controlling polyadenylation efficiency and alternative PAS usage.

### Proteins regulating 3′end processing

The regulation of mRNA 3′end processing is mainly achieved by RBPs and other interacting proteins assembling at *cis*-acting regulatory RNA elements within a transcript (Tian & Manley, 2013). Some of these proteins, such as TFIID (transcription factor II D) (Dantonel *et al*, 1997), have previously been shown to also be involved in other steps of RNA biogenesis, like transcription, splicing or constitutive 3′end processing. Others, including the tumour suppressor p53 (Nazeer *et al*, 2011) and the heat shock factor protein HSF1 (Xing *et al*, 2004), have previously been documented to contribute to completely unrelated annotated cellular functions, such as protein stability, stress response or RNA degradation (supplementary Table S1).

It has been shown that the cellular levels of core 3′end processing factors play a role in regulated 3′end processing. Single knockdown experiments of both the subunits of human cleavage factor CFIm (CFIm 25 and CFIm 68) resulted in a transcriptome-wide shift towards the use of proximal PASs, revealing a role of CFIm in APA (Gruber *et al*, 2012; Kubo *et al*, 2006; Martin *et al*, 2012; Sandberg *et al*, 2008). Further, isoforms of CstF-64 are associated with APA in different cell types, such as T-effector cells and male germ cells (Chennathukuzhi *et al*, 2001; Chuvpilo *et al*, 1999; Dass *et al*, 2007; Hockert *et al*, 2011; Monarez *et al*, 2007; Shell *et al*, 2005; Yao *et al*, 2012). Specifically, CstF-64 levels influence the class switch of immunoglobulin heavy chain synthesis (Takagaki *et al*, 1996). Finally, PAS usage has been recently shown to be affected by nuclear poly(A)-binding protein 1 (PAPBN1) levels. Biochemical studies demonstrated that PAPBN1 binds to poly(A) sites and that the depletion of PAPBN1 increases the usage of more upstream poly(A) sites (de Klerk *et al*, 2012; Jenal *et al*, 2012). Recent work suggests that PAPBN1 may influence PAS selection by directly binding to and thus inhibiting promoter-proximal PAS (Jenal *et al*, 2012). However, other findings propose that PAPBN1 preferentially binds to canonical PAS and stimulates 3′end processing (de Klerk *et al*, 2012). The exact underlying molecular mechanism(s) by which PAPBN1 affects poly(A) site usage remains to be elucidated.

3′End processing of pre-mRNAs is strongly interconnected with other steps of RNA biogenesis. Splicing factors, *e.g*. hnRNPs, and transcription-associated proteins, including the transcription factor E2F, have been found to regulate mRNA 3′end formation (supplementary Table S1). This is exemplified by hnRNPI/PTB1, which is a well-known global splicing repressor when bound to polypyrimidine tracts. hnRNPI/PTB1 can act as a regulator of 3′end processing efficiency upon binding to *cis*-acting RNA sequence elements playing a role in polyadenylation, such as the upstream sequence element (USE) and the DSE (Blechingberg *et al*, 2007b; Castelo-Branco *et al*, 2004; Danckwardt *et al*, 2011; Hall-Pogar *et al*, 2007; Millevoi *et al*, 2009). Likewise, the basal splicing factor U2AF65 can influence the functionality of the 3′end processing machinery by assembling at different polyadenylation-associated RNA elements, including the USE. U2AF65 can directly interact with core 3′end processing factors, including PAP, and can regulate the efficiency of 3′end processing by competing with other RBPs that assemble at such elements (Danckwardt *et al*, 2011; Hall-Pogar *et al*, 2007; Ko & Gunderson, 2002; Millevoi *et al*, 2006). Furthermore, the transcription factors TFIID and E2F can directly or indirectly affect mRNA polyadenylation either by binding to core 3′end processing factors or by stimulating their expression (Dantonel *et al*, 1997; Elkon *et al*, 2012; Martincic *et al*, 2009; Nagaike *et al*, 2011).

## Regulated 3′end processing and its impact on pathophysiology

During the last decade, biomedical research has revealed how regulated 3′end processing influences important physiological pathways and how misregulated mRNA 3′end processing can cause human disease (Danckwardt *et al*, 2008; Elkon *et al*, 2013). Below, we describe the pathophysiological impact of misregulated mRNA 3′end processing in detail.

### Physiological consequences of regulated 3′end processing

#### Quantitative regulation of gene and protein expression

**Figure 3:**
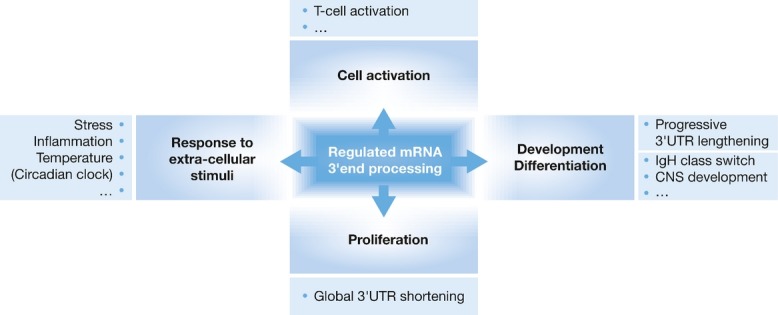
The control of 3′end processing post-transcriptionally adjusts mRNA and protein levels in response to environmental conditions, such as stress or inflammation. Further, it plays a role in development and differentiation, as in the case of the immunoglobulin heavy chain class switch and in the development of the central nervous system (CNS). In addition, alternative 3′end processing functions as a regulatory mechanism upon cell activation, *e.g*. T-cell activation, and proliferation. While a progressive lengthening of 3′UTRs via APA is typically observed during development and differentiation, higher proliferation states are associated with global 3′UTR shortening.

By controlling the efficiency of the 3′end processing reaction, mRNA and protein levels can be post-transcriptionally adjusted according to cellular needs in response to cellular signalling or environmental conditions. As described above, this is achieved via auxiliary *cis*-acting RNA elements and regulatory *trans*-acting proteins, which can either stimulate or inhibit the utilization of PASs in response to exogenous stimuli (Fig 3). As a consequence, the amount of mature mRNA and protein is altered appropriately, thus modulating physiological processes. First described for viral RNA, the USE has subsequently been identified to stimulate the efficiency of 3′end processing in mammalian mRNAs (Gilmartin *et al*, 1995; Graveley *et al*, 1996). The prothrombin RNA represents the prototype for the function of the USE (Danckwardt *et al*, 2007). Prothrombin is the zymogen of the serine protease thrombin and is one of the key proteins of blood coagulation, complement activation, angiogenesis and tumour invasion. The expression of prothrombin is tightly regulated in a USE-dependent fashion in response to inflammation and stress (Danckwardt *et al*, 2011), as already a small inappropriate increase by less than twofold gives rise to thrombophilia, a predisposition to develop thrombosis (Gehring *et al*, 2001). Normally, the USE is occupied by the inhibitory proteins FBP2 and FBP3. Following inflammation or other stress stimuli, p38MAPK is activated leading to the phosphorylation of these two proteins and their concomitant release from the USE. The USE then becomes available for the binding of the 3′end processing activator proteins U2AF35, U2AF65 and hnRNPI/PTB1. This results in elevated prothrombin mRNA and protein synthesis and the stimulation of prothrombin downstream pathways (Danckwardt *et al*, 2011). A similar USE-regulated mechanism of gene expression has been found in several other mRNAs, namely those encoding the complement factor C2, the collagen genes COL1A1, COL1A2 and COL2A1, lamin B2 and the cyclooxygenase COX-2 (Brackenridge & Proudfoot, 2000; Hall-Pogar *et al*, 2007; Moreira *et al*, 1998; Natalizio *et al*, 2002). The USE system thus exemplifies how regulated mRNA 3′end processing can post-transcriptionally contribute to the fine-tuning of physiological processes in response to external stimuli.

Although not yet understood in its mechanistic details, the expression Hsp70.3 is another example of the importance of regulated 3′end processing. Thermal stress leads to alternatively polyadenylated transcripts with shortened 3′UTRs, potentially lacking miRNA binding, which are more stable and associated with an increased biosynthesis of the Heat shock protein 70 (Hsp70) (Tranter *et al*, 2011). Moreover, the polyadenylation of a set of different mRNAs is catalysed by an inducible non-canonical poly(A) polymerase, termed STAR-PAP (nuclear speckle targeted PIPKIα regulated-poly(A) polymerase). STAR-PAP activity can be stimulated by stress-induced signalling pathways. Interestingly, this non-canonical poly(A) polymerase controls the expression of mRNAs encoding proteins associated with cellular stress response and disease, including cancer (Gonzales *et al*, 2008; Laishram *et al*, 2011; Li *et al*, 2013; Mellman *et al*, 2008).

Apart from the quantitative regulation of the expression of specific (sets of) genes via stimulated 3′end processing, the transcriptome-wide modulation of polyadenylation efficiency has been reported in response to various stress conditions, including thermal stress and DNA damage (Nazeer *et al*, 2011; Xing *et al*, 2004). Interestingly, thermal stress or DNA damage have been demonstrated to globally inhibit polyadenylation efficiency, whereas specific stress response genes are not affected by this repression (Di Giammartino *et al*, 2013; Nazeer *et al*, 2011). Taken together, regulated 3′end processing contributes to the maintenance of important physiological pathways and specifically appears to play a role in protective responses of cells to exogenous stimuli, such as stress.

#### Qualitative regulation of gene and protein expression via alternative polyadenylation

Physiological processes can be further influenced by APA that triggers the expression of different transcript or protein isoforms (Fig 3).

#### Development

The importance of APA in cellular differentiation was first demonstrated when Brown and Morrison reported that the immunoglobulins heavy chain (IgH) class shift is triggered by APA (Brown & Morrison, 1989). The production of the secreted or the membrane bound form of immunoglobulins, respectively, is achieved via a splicing-dependent poly(A) site switch in IgH genes which is based on a competition between splicing and 3′end processing (Peterson, 2007). While developmentally “younger” B cells express immunoglobulins in a membrane-bound form on their cell surface, the more mature plasma cells produce immunoglobulins as secreted proteins, which is mediated by the usage of a PAS located further 5′ (Peterson, 2011).

The role of regulated 3′end processing during development and differentiation was extended by the analysis of the time-dependent protein expression program of GFAP (glial fibrillary acidic protein) in the central nervous system (CNS). GFAP forms the intermediate cytoskeleton in mature astrocytes. APA results in the dynamic expression of two GFAP protein isoforms (GFAPɛ and GFAPκ) (Blechingberg *et al*, 2007b). The GFAPκ/ɛ-ratio has been found to decrease during cortex development suggesting that APA of this pre-mRNA plays a pivotal role in CNS development (Blechingberg *et al*, 2007a). Biochemical analyses suggest a role for splicing factors, such as hnRNPI/PTB1 and SR-proteins, in triggering the APA-dependent GFAP isoform switch but the exact underlying molecular mechanism remains to be elucidated (Blechingberg *et al*, 2007b).

Development-associated APA can also trigger the expression of mRNAs that differ in 3′UTR-length, leading to the exclusion or inclusion of miRNA binding sites and a change in the regulatory state of a specific transcript. This is exemplified by PAX3, a key regulator of myogenesis during development. PAX3 promotes proliferation, inhibits differentiation and is transiently expressed during the activation of adult muscle stem cells. In quiescent stem cell populations, its expression is usually suppressed via miR-206 that has been found to be highly abundant in these cells. In specific muscles, such as in the diaphragm muscle, PAX3 is able to escape the miRNA-driven regulation in quiescent muscle stem cells. This is due to 3′UTR shortening via APA at a more 5′PAS (Boutet *et al*, 2012). These observations predict a role of regulated 3′end processing in controlling stem cell function and implicate APA as a powerful mechanism to modulate miRNA-dependent regulation of gene expression during development.

More generally, APA functions as a regulatory mechanism on a transcriptome-wide scale in response to environmental demands by changing the regulatory state of mRNAs. Highly parallel RNA sequencing methods have recently helped to gain insights into the global modulation of mRNA 3′end processing. In the course of development and differentiation, regulated 3′end processing can function in a tissue-specific fashion, such as triggering APA during spermatogenesis or in the brain (Dass *et al*, 2007; Hockert *et al*, 2011; Li *et al*, 2012; Liu *et al*, 2007; Miura *et al*, 2013). The utilization of promoter-distal PASs generally increases with the developmental and differentiation state, and mRNAs with longer 3′UTRs generally contain a higher number of miRNA and protein binding sites (Hoque *et al*, 2013; Shepard *et al*, 2011). During later stages of development, mRNAs thus tend to be subject to more complex mechanisms of post-transcriptional control.

#### Proliferation and cell activation

In contrast to the progressive lengthening of 3′UTRs observed during development and differentiation, the usage of promoter-proximal PASs and therefore diminished regulation via miRNAs or RBPs is linked to proliferation (Elkon *et al*, 2012; Sandberg *et al*, 2008). Not only do proliferating cells exhibit significantly shortened 3′UTRs when compared to cells in a resting state, but are also subject to enhanced cleavage at intronic PAS. Members of the E2F family which influence cell cycle progression have been found to stimulate the expression of 3′end processing factors and proximal poly(A) site usage in proliferating cells (Elkon *et al*, 2012).

Further, APA has been shown to play a role in cell activation. This is illustrated by the inducible expression of NF-ATc (nuclear factor of activated T cells) upon antigen exposure. The stimulation of the T-cell antigen receptor triggers a switch from long protein isoforms in naïve T cells to a short isoform in mature T cells. Whereas in naïve T cells, the “weak” proximal PAS is not efficiently used, mature T cells show proximal poly(A) site usage and the short NF-ATc isoform is produced. The short NF-ATc isoform stimulates the expression of T-cell-specific genes, including lymphokine and cytokine genes (Chuvpilo *et al*, 1999; Serfling *et al*, 2000). Moreover, when comparing transcript expression in different immune cells (T lymphocytes, B cells, monocytes) before and after stimulation, a decrease of the expression of longer isoforms, which are processed at more distal PASs, has been reported to be associated with higher proliferation states (Sandberg *et al*, 2008). A transcriptome-wide shift towards promoter-proximal PAS usage has also been found in activated neurons (Flavell *et al*, 2008). Recent studies suggest a role of U1snRNP in APA. Low U1snRNP levels in activated neuronal cells have been shown to lead to widespread shortening of mRNAs due to a shift to proximal PASs, and that U1snRNP overexpression can inhibit this effect (Berg *et al*, 2012). Nonetheless, the molecular mechanism underlying U1snRNP-dependent APA is still unknown.

#### Cold shock and circadian gene expression

mRNA polyadenylation can be qualitatively regulated in response to cold shock and to the circadian clock. Two cold-induced RBPs, CIRBP and RBM3, have been found to repress promoter-proximal poly(A) site usage in a set of genes under cold shock conditions, leading to elongated 3′UTRs (Liu *et al*, 2013). Interestingly, further investigations revealed that many of the affected genes show 3′UTR-lengthening or -shortening via APA with strong circadian oscillations depending on the ambient temperature. These findings suggest that APA contributes to the regulation of the temperature-dependent circadian clock.

### Human pathology associated with altered 3′end processing

**Figure 4:**
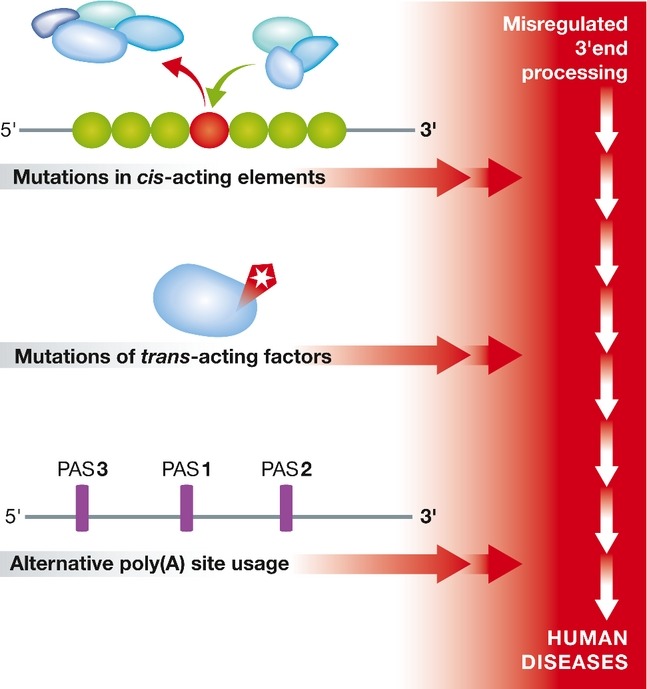
Misregulation of mRNA 3′end processing can be caused by constitutive errors in*cis*-acting RNA elements, mutations of *trans*-acting proteins or changes in poly(A) site usage of otherwise normal transcripts. Mutations in RNA sequence elements or *trans*-acting proteins both potentially interfere with the interaction of RBPs and the RNA, leading to gain or loss of function. APA triggers the production of mRNAs varying in the length of their 3′UTR or in their C-terminal coding region, which leads to the expression of different protein isoforms. Certain diseases, such as cancer, are characterized by a polyadenylation pattern significantly different from the one in a healthy state.

Errors in mRNA processing leading to human disease can be caused by mutations in RNA sequence elements with a critical role in mRNA 3′end processing, defects in the biological function of *trans*-acting protein factors, or differential usage of PASs within otherwise normal transcripts (Fig 4).

### Mutations in *cis*-acting elements

#### Haematologic diseases

The importance of 3′end processing for normal gene function and human health was first illustrated by an inactivating mutation in the hexamer of the poly(A) signal (AATAAA → AATAAG) of the α 2-globin gene causing α-thalassaemia (Higgs *et al*, 1983). Subsequently, other mutations affecting the α-globin and the β-globin polyadenylation signal were found in patients with α- and β-thalassaemia (Harteveld & Higgs, 2010). Interestingly, human disease cannot only result from the inactivation of efficient 3′end processing but also from pathological gain-of-function-mutations. This has first been demonstrated by a commonly occurring mutation of the CS of the prothrombin pre-mRNA, with a prevalence of heterozygotes of 1.7–3% in the Caucasian population (Gehring *et al*, 2001; Rosendaal *et al*, 1998). Physiologically, cleavage occurs at a suboptimal CG dinucleotide. A CG → CA mutation (G20210A) at this position transforms the physiological, inefficient site into a more active site thus increasing prothrombin synthesis. Because prothrombin is one of the key pro-coagulatory proteins of the network controlling blood coagulation, this mutation results in thrombophilia, *i.e*. a predisposition to develop thrombosis.

#### Cancer

Back in the 1860s, Armand Trousseau was the first to connect a prothrombotic state to cancer (Trousseau, 1865). This early finding foreshadowed an association of regulated 3′end processing with more complex diseases by some 150 years. Recently, mutations in *cis*-acting RNA elements of ubiquitously expressed genes leading to defective 3′end processing have indeed been linked to cancer. The tumour suppressor gene p53, a crucial regulator of the cell cycle and apoptosis, is mutated in approximately 60% of human cancers (Muller & Vousden, 2013). A germline mutation in the p53 polyadenylation signal (AAUAAA → AAUACA), which occurs in approximately 0.5–2% of different European populations, has been reported to predispose to basal cell carcinoma, prostate cancer, glioma and colorectal cancer (Enciso-Mora *et al*, 2013; Stacey *et al*, 2011).

#### Other disorders resulting from PAS mutations

Mutations of mRNA 3′end processing elements are further known to cause complex syndromes. An example is a polyadenylation signal mutation (AAUAAA → AAUGAA) in the FOXP3 (forkhead box P3) gene causing the IPEX syndrome, which is characterized by immune dysfunction, polyendocrinopathy and enteropathy (Bennett & Ochs, 2001). The mutation leads to a very long 3′UTR of the FOXP3 mRNA, which becomes relatively unstable, thus resulting in decreased FOXP3 protein levels. FOXP3 functions as a transcription factor in regulatory T cells, explaining the immune dysfunction of affected patients.

In addition, panic disorders and altered fear extinction memory have been associated with a common mutation in the distal polyadenylation signal of the serotonin (5-hydroxytryptamine) transporter gene (SERT or 5-HTT) (Gyawali *et al*, 2010; Hartley *et al*, 2012). This mutation leads to the usage of the more proximal PAS and to the expression of a transcript with a shortened 3′UTR and a potentially higher stability. These findings associate the PAS polymorphism occurring in the serotonin transporter gene with mood and anxiety disorders in humans.

### Mutations of *trans*-acting factors

Defects in *trans*-acting proteins with a role in 3′end processing appear to be rare. An example of such an error is a triplet-repeat expansion of PABPN1 (trePABPN1) that causes autosomal-dominant oculopharyngeal muscle dystrophy (OPMD). trePABPN1 interacts with WT PABPN1 in a dominant negative fashion and interferes with its function as a suppressor of proximal poly(A) site usage. Mouse models of OPMD as well as human cells expressing trePABPN1 show a global shift towards proximal, usually “weaker” PAS (de Klerk *et al*, 2012; Jenal *et al*, 2012). How exactly this change in poly(A) site usage causes the phenotype of OPMD remains to be investigated.

### Diseases caused by alternative polyadenylation

Differential poly(A) site usage in otherwise normal transcripts is associated with malignancies. A number of diseases have been linked to either APA of single transcripts or to global changes in polyadenylation. The symptoms of such diseases include growth retardation, cancer predisposition syndromes and neurodegeneration.

#### Cancer predisposition syndromes

Lynch syndrome is a hereditary predisposition to several types of cancer, including colorectal and endometrial cancer, which is caused by a loss of function of one of the four DNA mismatch repair genes MSH2, MLH1, MSH6, PMS2. Some patients with this syndrome carry germline mutations in the coding region of one of those genes. Recently, a 20 nucleotide duplication in close vicinity to the poly(A) signal of MSH6 mRNA has been detected in two patients, associated with decreased MSH6 mRNA levels (Decorsiere *et al*, 2012). Although the underlying molecular mechanism is still unknown, the duplication has been found to negatively affect polyadenylation efficiency (Decorsiere *et al*, 2012). These findings link decreased MSH6 mRNA expression caused by impaired mRNA 3′end processing to Lynch syndrome.

#### Neurodegenerative diseases

The development of the central nervous system is a complex process, involving tight regulation of gene expression. The fragile X syndrome is the most common form of inherited intellectual disability. This disease is caused by the methylation and the expansion (>200 repeats) of a CGG element within the promoter region of the fragile X mental retardation 1 (FMR1) gene, with the premutation (55–200 CGG repeats) already showing a clinically relevant phenotype (McLennan *et al*, 2011). Several isoforms of the FMR1 mRNA are generated via alternative splicing, as well as via APA (Tassone *et al*, 2011; Verkerk *et al*, 1993). The analysis of brain specimen of CGG knock-in mice and of post-mortem brain tissues from a carrier of the premutation revealed differential usage of poly(A) sites within the FMR1 3′UTR when compared to control samples (Tassone *et al*, 2011). Together with observed variations in the 5′UTR, this poly(A) site choice likely contributes to the pathology of the fragile X syndrome.

#### Cancer

In recent years, alterations in cleavage and polyadenylation of single genes have been linked to the development and progression of numerous different forms of cancer. Specifically, tumour cell lines and primary human cancer material from patients with mantle cell lymphoma (MCL) exhibited different polyadenylation patterns than control cells or tissues. MCL is related to the APA of cyclin D1. In proliferating MCL tumours, truncated cyclin D1 mRNA isoforms originating from processing at a proximal PAS are over-represented (Mayr & Bartel, 2009; Wiestner *et al*, 2007). Although the underlying molecular mechanism triggering the APA of cyclin D1 mRNA is unknown, the shorter transcript variants have been found to be more stable than their full-length counterparts, which may be attributable to a loss of miRNA-driven degradation. The increased mRNA stability leads to elevated Cyclin D1 protein expression and correlates with increased overall survival of patients (Rosenwald *et al*, 2003). Strikingly, cancer has been also associated with APA-dependent shortening of mRNAs observed on a transcriptome-wide scale. High-throughput sequencing methods revealed a global shift towards promoter-proximal poly(A) site usage in tumour cell lines as well as in tumour samples derived from different organs, such as breast, colon, kidney, liver and lung, which might contribute to the stabilization and/or translational activation of mRNAs encoding oncogenes and other cancer-relevant proteins (Lin *et al*, 2012; Mayr & Bartel, 2009).

## 3′End processing on its way to the clinic

Based on the current knowledge about regulated 3′end processing in human pathophysiology, it seems reasonable to consider its potential role in diagnosis and as a therapeutic target in the future. High-throughput methods used to quantitatively and qualitatively profile RNA PASs on a global scale revealed tissue-specific polyadenylation patterns suggesting coordinated regulation of these processes (Wang *et al*, 2008). Furthermore, there are population genetic variations of mRNA 3′end processing in general and in poly(A) site usage in particular (Wang *et al*, 2008; Yoon *et al*, 2012; Zhang *et al*, 2005). Inter-individual differences in 3′end architecture and, therefore, in the regulation of 3′end processing, have been found to cause the observed changes in mRNA polyadenylation and cleavage between individuals, contributing to “personal” gene expression profiles. These observations suggest that such variations may modulate medically relevant phenotypes. As described above, samples derived from numerous forms of cancer exhibit polyadenylation profiles that significantly differ from samples derived from healthy tissue (Fu *et al*, 2011; Lin *et al*, 2012; Mayr & Bartel, 2009; Morris *et al*, 2012). Genes undergoing cancer-associated 3′UTR shortening or, in rarer cases, elongation, can be clustered according to functional groups and categories, foreshadowing their potential role as significant biomarkers, which may help to distinguish tumour subtypes with distinct molecular signatures, thus signifying biological heterogeneity which potentially impacts on treatment response and prognosis (Morris *et al*, 2012).

Apart from using 3′end processing profiles as diagnostic tools, the polyadenylation step might serve as a target of future medical therapies. Since mRNA 3′end processing is regulated via *trans*-acting proteins and *cis*-acting RNA sequence elements, successful therapies must aim at this interplay. Inhibitors as well as activators of the proteins involved in mRNA polyadenylation have been shown to have the potential to treat 3′end processing-associated diseases in model systems. This is exemplified by the anti-inflammatory effect of cordycepin (3′ deoxyadenosine, dATP), a compound that inhibits polyadenylation by arresting the cleavage complex (Kondrashov *et al*, 2012). Interestingly, dATP treatment was reported to inhibit the 3′end processing of inflammatory genes without affecting the polyadenylation of control transcripts, indicating drug specificity for gene-specific differences in the 3′end processing step. Importantly, the gene-specific differences of the polyadenylation process provide the perspective to develop therapies targeting the 3′end processing machinery of distinct genes and the associated pathologies. Specifically, the interactions of *trans*-acting proteins with their cognate RNA binding motifs such as those that have been reported between the USE and stimulatory and inhibitory proteins of 3′end processing (Danckwardt *et al*, 2011) could, in principle, be envisaged to be targeted by small molecules thus modulating 3′end processing efficiency of specific classes of genes.

In addition, directly modulating the function of RNA molecules in mammalian cells via the introduction of antisense oligonucleotides has gained much attention as a potential therapeutic strategy (Bennett & Swayze, 2010). Antisense oligonucleotides that inhibit PASs have been successfully used to modulate poly(A) site selection in cultured cells (Vickers *et al*, 2001). Potentially, targeting *cis*-acting elements playing a role in the cleavage and polyadenylation reaction might help to treat associated diseases in the future.

## Conclusions and future perspectives

The regulation of mRNA 3′end processing has gained much attention in recent years as a mechanism that can fine-tune gene expression in a quantitative and qualitative manner. Its role in pathophysiology and the underlying molecular mechanisms are intensively investigated at present. Certainly, the number of physiological and pathological cellular pathways controlled via regulated mRNA polyadenylation and cleavage will progressively increase. Prospectively, regulated mRNA 3′end processing could instruct new diagnostic approaches and serve as a therapeutic target, aiding in treating diseases, including cancer.

## Conflict of interest

The authors declare that they have no conflict of interest.

**Pending issues**

Develop strategies to therapeutically target misregulated mRNA 3′end processing.

Unravel the molecular mechanisms underlying misregulated mRNA 3′end processing to increase the level of understanding of its impact on pathological and physiological pathways.

Explore the differences in mRNA 3′end processing as a diagnostic tool.

## Abbreviations

mRNA 3′end processing: Cleavage of pre-mRNA and its subsequent polyadenylation, ensured by a highly regulated network of proteins in the nucleus.
3′UTR: Untranslated region at the 3′end of an mRNA molecule that contains regulatory RNA sequence elements, including the polyadenylation signal, miRNA- and RBP-binding sites.
*Trans*-acting: Acting from a different molecule (*e.g*. protein regulates RNA).
*Cis*-acting: Acting from within the same molecule (*e.g*. RNA sequence element controls the RNA it is contained in).
Germline mutation: Any variation in cells that differentiate into gametes and somatic cells.
Thalassaemia: A group of diseases caused by mutations of the α- or β-globin genes leading to decreased production of globin chains and severe anemia.
Oculopharyngeal muscle dystrophy (OPMD): Autosomal dominant neuromuscular disease that is caused by a mutation in the *PABPN1* gene. It is characterized by ptosis (droopy eyelids), muscle weakness and difficulty in swallowing (dysphagia).
Antisense oligonucleotides: Oligonucleotides that can bind to RNA through Watson-Crick base pairing and can modulate the function of the target RNA
