# Supplementary material for: mRNA 3′end processing: A tale of the tail reaches the clinic
Source: EMBO Mol Med. 2013 Oct 9;6(1):16–26. doi: 10.1002/emmm.201303300 (PMC3936486; doi:10.1002/emmm.201303300)
Supplement: Supplementary file 1 [file emmm0006-0016-sd1.pdf]

Supplementary table S1

Proteins involved in mRNA 3'end processing				
**Cellular function**	**Mammalian factor**		**Function in 3'end processing**	**Reference**
**Constitutive 3'end processing**				
**RNA Cleavage**				
	**Cleavage stimulation factor** (CstF)			
		CstF77	Cleavage, dimerization	(Takagaki et al, 1990)
		CstF64/τ	Cleavage	(Shi et al, 2009; Takagaki et al, 1990; Wallace et al, 1999)
		CstF50	Cleavage, dimerization	(Takagaki et al, 1990)
	**Cleavage factor I** (CFIm)			(Awasthi & Alwine, 2003; Ruegsegger et al, 1996; Ruegsegger et al, 1998)
		CFIm 25	Cleavage, dimerization	(Coseno et al, 2008; Dettwiler et al, 2004; Ruegsegger et al, 1996)
		CFIm 59/68/ 72	Cleavage	(Brown & Gilmartin, 2003; Ruegsegger et al, 1996)
	**Cleavage factor II** (CFIIm)			(de Vries et al, 2000)
		hPcf11	Transcription termination	(Shi et al, 2009)
		hClp1	RNA 5' kinase	(de Vries et al, 2000; Haddad et al, 2012)
**RNA cleavage & polyadenylation**				
	**Cleavage and polyadenylation specificity factor** (CPSF)			
		CPSF160	Polyadenylation	(Bienroth et al, 1991; Keller et al, 1991; Murthy & Manley, 1995)
		CPSF100	Endonuclease?	(Jenny et al, 1994; Kyburz et al, 2006)
		CPSF73	Endonuclease	(Mandel et al, 2006)

		CPSF30		(Barabino et al, 1997; Bienroth et al, 1991)
		hFip1	PAP stimulation	(Kaufmann et al, 2004)
		Symplekin	Cleavage, scaffolding	(Takagaki & Manley, 2000)
		Wdr33	Scaffolding	(Shi et al, 2009)
**RNA polyadenylation**				
	**Poly(A) Polymerases**			
		PAP	Poly(A) Polymerase	(Takagaki et al, 1988)_ENREF_33
		Neo-PAP/ PAPOLG	Poly(A) Polymerase	(Topalian et al, 2001)
		Star-PAP	Poly(A) Polymerase (specific mRNAs)	(Mellman et al, 2008)
		TPAP (PAPβ)	Testis-specific poly(A) polymerase	(Kashiwabara et al, 2000)
		PAPγ	Nuclear poly(A) polymerase	(Kyriakopoulou et al, 2001)
	**Retinoblastoma binding protein** (Rbbp6)		Linking 3'end processing-tumorigenesis	(Shi et al, 2009)
	**Protein phosphatase 1 α/β** (PP1α/β)		Serine/threonine phosphatase	(Shi et al, 2009)
**Polyadenylation, mRNA export, translation**				
	**Poly(A)-binding proteins (PABP)**			
		PABPN1	Poly(A) tail length control, PAP stimulation	(Wahle, 1991)
		PABPC(1,2,4,5)	Poly(A) binding	(Blanco et al, 2001; Feral et al, 2001; Grange et al, 1987; Yang et al, 1995)
**Other components of the 3'end processing complex**				
	**RNA polymerase II-associated factor complex** (PAF)			(Shi et al, 2009)
	**Facilitates chromatin transcription complex** (FACT)			(Shi et al, 2009)

	**DNA-damage-response factors**	DNA-PK, Ku 70, Ku 86/XRCC5, Rpb11		(Shi et al, 2009)
	**Transcription factors**	TFII/I, TAF15		(Shi et al, 2009)
	**Splicing factors**	SF3A, SF3A60, SF3B, Prpf38b, SRm300, U1 70K, U2AF, PRP19, p54nrb, PSF, PUF60, UAP56, SF1		(Shi et al, 2009)
	**Exosome**	SKIV2L2 (hMTR4)		(Shi et al, 2009)
	**Translation factors**	eEF1-α/-γ, eIF2A, eIF4A, eIF3- γ, eIF3S2/5/6/9S, eIF3A, eIF4G1, RACK1/GNB2L1 /lung cancer oncogene 7		(Shi et al, 2009)
	**DEAD box polypeptides**			(Shi et al, 2009)
	**DEAH box polypeptides**			(Shi et al, 2009)
	**Integrator complex** (INT)	INTS2/3/4/6/7/8/9/10		(Baillat et al, 2005; Shi et al, 2009)_ENREF_7
	**RNA binding motif proteins**			(Shi et al, 2009)
	**Zinc finger, CCHC domain containing 8** (ZCCHC8)			(Shi et al, 2009)
	**EBNA2 coactivator p100** (SND1)			(Shi et al, 2009)
	**Gamma-catenin** (JUP)			(Shi et al, 2009)
	**Ras-GTPase-activating protein SH3-domain-binding protein** (G3BP)			(Shi et al, 2009)
	**Protein phosphatase 1 nuclear-targeting subunit** (PNUTS)			(Shi et al, 2009)
	**Interferon-induced protein with tetratricopeptide repeats** (IFIT)			(Shi et al, 2009)

**Regulated 3'end processing**				
**Core 3’end processing factors**				
	**CFIm**		APA	(Gruber et al, 2012; Sartini et al, 2008)
		CFIm 25	APA	(Kubo et al, 2006)
		CFIm 59/68/72	quantitative regulation of 3'end processing, APA	(Brown & Gilmartin, 2003; Martin et al, 2012; Venkataraman et al, 2005)
	**PABPN1**		PAP stimulation, APA, poly(A) length control	(de Klerk et al, 2012; Jenal et al, 2012)
	**Rbbp6**		Linking 3'end processing-tumorigenesis	(Shi et al, 2009)
	**CstF64/τ**		(Tissue-specific) APA	(Chennathukuzhi et al, 2001; Chuvpilo et al, 1999; Dass et al, 2007; Hockert et al, 2011; Li et al, 2012; Shell et al, 2005; Shi et al, 2009; Takagaki et al, 1996) _ENREF_7
**Transcription-associated factors**				
	**CTD (RNAP II)**		Linking 3'end processing-transcription polyadenylation inhibition	(Hirose & Manley, 1998; McCracken et al, 1997)
	**Transcription factor II D** (TFIID)		CPSF recruitment	(Dantonel et al, 1997)
	**Bromodomain testis-specific protein** (BRDT)		3'-UTR processing regulation in round spermatids	(Berkovits et al, 2012)
	**Elongation factor, RNA polymerase II, 2** (ELL2)		APA	(Martincic et al, 2009)
	**Mediator complex subunit 23** (MED23)		APA	(Huang et al, 2012)
	**E2F**		Global stimulation of genes encoding 3'-end processing proteins in proliferation	(Elkon et al, 2012)
**Transcription-/ nuclear export-associated factors**				
	**THO complex 5** (THOC5)		APA	(Katahira et al, 2013)

**Splicing factors**				
	**heterogeneous nuclear ribonucleoprotein I** (hnRNPI)		Quantitative regulation of 3'end processing, APA	(Blechingberg et al, 2007; Castelo-Branco et al, 2004; Danckwardt et al, 2011; Danckwardt et al, 2007; Hall-Pogar et al, 2007; Lou et al, 1999; Millevoi et al, 2009; Moreira et al, 1998)_ENREF_3
	**heterogeneous nuclear ribonucleoprotein L** (hnRNPL)		APA	(Hung et al, 2008)
	**heterogeneous nuclear ribonucleoprotein H/H'** (hnRNP H/H')		Polyadenylation stimulation	(Alkan et al, 2006; Bagga et al, 1998; Dalziel et al, 2007; Millevoi et al, 2009)
	**heterogeneous nuclear ribonucleoprotein F** (hnRNP F)		Cleavage inhibition	(Veraldi et al, 2001)
	**U2 auxiliary factor** (U2AF)	U2AF35	3'end processing stimulation	(Danckwardt et al, 2011; Danckwardt et al, 2007; Hall-Pogar et al, 2007)_ENREF_3
		U2AF65	Quantitative regulation of 3'end processing, PAP inhibition, poly(A) length control	(Danckwardt et al, 2011; Danckwardt et al, 2007; Hall-Pogar et al, 2007; Millevoi et al, 2006; Vagner et al, 2000b)
	**Ribo-nucleoprotein PTB-binding 1** (RAVER1)		Quantitative regulation of 3'end processing?	(Danckwardt et al, 2011; Danckwardt et al, 2007; Gromak et al, 2003; Hall-Pogar et al, 2007)
	**KH-Type Splicing Regulatory Protein** (FBP2, KHSRP)		3'end processing inhibition	(Danckwardt et al, 2011)
	**Far upstream element-binding protein 3** (FBP3)		3'end processing inhibition	(Danckwardt et al, 2011)
	**CUG repeat binding protein 1** (CUGBP1)			(Danckwardt et al, 2011)
	**Translational repression by RNA-binding protein** (TIAR)			(Danckwardt et al, 2011)
	**T-cell-restricted intracellular antigen-1** (TIA1)			(Danckwardt et al, 2011)
	**U1 small nuclear ribonucleoproteins**	U1 snRNP	Quantitative regulation of 3'end processing, APA, shielding of cryptic poly(A) sites	(Andersen et al, 2012; Awasthi & Alwine, 2003; Berg et al, 2012; Gunderson et al, 1998; Hall-Pogar et al, 2007; Kaida et al, 2010; Lou et al, 1998; Lutz et al, 1996; Vagner et al, 2000a)
		U1A, U1A snRNP	Quantitative regulation of 3'end processing	(Boelens et al, 1993; Gunderson et al, 1998; Hall-Pogar et al, 2007;

				Lutz & Alwine, 1994; Lutz et al, 1996; Phillips et al, 2004)
	**U2 small nuclear ribonucleoproteins** (SF3 subunits)		Linking splicing to 3′-end processing	(Kyburz et al, 2006)
	**54-kDa nuclear RNA-binding protein** (p54nrb)		3'end cleavage stimulation	(Hall-Pogar et al, 2007; Kaneko et al, 2007; Liang & Lutz, 2006)
	**p54nrb–protein-associated splicing factor** (PSF)		Transcription-dependent stimulation of pre-mRNA processing	(Rosonina et al, 2005)
	**Neuronal RNA binding protein** (Nova)		Quantitative regulation of 3'end processing	(Licatalosi et al, 2008)
	**Serine/arginine-rich proteins**	SRp20	APA	(Lou et al, 1998)
		SRp75	PAP and polyadenylation inhibition	(Ko & Gunderson, 2002)
		SRm160	3'end cleavage stimulation	(McCracken et al, 2003)
**Chaperones**				
	**14-3-3ε**		Polyadenylation inhibition	(Kim et al, 2003)
**Tumor suppressors**				
	**BRCA1/BARD1-complex** (breast cancer 1/BRCA1 associated RING domain 1)		3'end processing inhibition	(Edwards et al, 2008; Kim et al, 2006; Kleiman et al, 2005)
	**PAF complex (Cdc73)**		3'end processing stimulation, linking transcription to 3'end processing	(Rozenblatt-Rosen et al, 2009)
	**p53**		3'end processing inhibition	(Nazeer et al, 2011)
**3'-exoribonuclease**				
	**Poly(A)-specific ribonuclease** (PARN)		3′ cleavage inhibition under DNA-damaging conditions	(Cevher et al, 2010)
**Kinases**				
	**Polo**		Auto-regulation via APA	(Pinto et al, 2011)
**Other enzymes**				

	**Poly(ADP-ribose)-Polymerase 1** (PARP1)		PAP and polyadenylation inhibition upon heat shock	(Di Giammartino et al, 2013)
	**Ubiquitin specific peptidase 22** (USP22)		3'-end processing regulation of JAK-STAT-inducible genes	(Chipumuro & Henriksen, 2012)
**Heat shock factor proteins**				
	**Heat shock factor protein 1** (HSF1)		3' processing stimulation	(Xing et al, 2004)
	**Cellular stress response 1** (CSR1)		CPSF73 inactivation, polyadenylation inhibition	(Zhu et al, 2009)
**Others**				
	**Cold-inducible RNA-binding protein** (CIRBP)		APA	(Liu et al, 2013)
	**ELAV-like protein 1** (HuR)		3'processing inhibition, auto-regulation via APA	(Dai et al, 2012; Danckwardt et al, 2011; Mansfield & Keene, 2012; Zhu et al, 2009)
	**Inositol 1,4,5-triphosphate (IP3) receptor-binding protein released with IP3** (IRBIT)		Controlling cytoplasmic/nuclear partitioning of Fip1, PAP inhibition	(Kiefer et al, 2009)
	**Leucine-rich PPR motif-containing protein** (LRPPRC)		Polyadenylation regulation in mitochondria	(Ruzzenente et al, 2012)
	**LRPPRC/SRA stem-loop interacting RNA binding protein complex** (LRPPRC/SLIRP)		Polyadenylation stimulation in mitochondria	(Chujo et al, 2012)
	**RNA Binding Motif Protein 3** (RBMP3)		APA	(Liu et al, 2013)
	**Suppressor of forked protein** (Su(f))		Auto-regulation via regulated 3'end processing	(Audibert & Simonelig, 1998)
	**TAR DNA-binding protein 43** (TDP-43)		Auto-regulation via regulated 3'end processing	(Avendano-Vazquez et al, 2012)
	**PolyC-binding protein** (αCP)		APA	(Ji et al, 2013)_ENREF_107

References

Alkan SA, Martincic K, Milcarek C (2006) The hnRNPs F and H2 bind to similar sequences to influence gene expression. The Biochemical journal 393**:** 361-371

Andersen PK, Lykke-Andersen S, Jensen TH (2012) Promoter-proximal polyadenylation sites reduce transcription activity. Genes & development 26**:** 2169-2179

Audibert A, Simonelig M (1998) Autoregulation at the level of mRNA 3' end formation of the suppressor of forked gene of Drosophila melanogaster is conserved in Drosophila virilis. Proceedings of the National Academy of Sciences of the United States of America 95**:** 14302-14307

Avendano-Vazquez SE, Dhir A, Bembich S, Buratti E, Proudfoot N, Baralle FE (2012) Autoregulation of TDP-43 mRNA levels involves interplay between transcription, splicing, and alternative polyA site selection. Genes & development 26**:** 1679-1684

Awasthi S, Alwine JC (2003) Association of polyadenylation cleavage factor I with U1 snRNP. RNA 9**:** 1400-1409

Bagga PS, Arhin GK, Wilusz J (1998) DSEF-1 is a member of the hnRNP H family of RNA-binding proteins and stimulates pre-mRNA cleavage and polyadenylation in vitro. Nucleic acids research 26**:** 5343-5350

Baillat D, Hakimi MA, Naar AM, Shilatifard A, Cooch N, Shiekhattar R (2005) Integrator, a multiprotein mediator of small nuclear RNA processing, associates with the C-terminal repeat of RNA polymerase II. Cell 123**:** 265-276

Barabino SM, Hubner W, Jenny A, Minvielle-Sebastia L, Keller W (1997) The 30-kD subunit of mammalian cleavage and polyadenylation specificity factor and its yeast homolog are RNA-binding zinc finger proteins. Genes & development 11**:** 1703-1716

Berg MG, Singh LN, Younis I, Liu Q, Pinto AM, Kaida D, Zhang Z, Cho S, Sherrill-Mix S, Wan L et al (2012) U1 snRNP determines mRNA length and regulates isoform expression. Cell 150**:** 53-64

Berkovits BD, Wang L, Guarnieri P, Wolgemuth DJ (2012) The testis-specific double bromodomain-containing protein BRDT forms a complex with multiple spliceosome components and is required for mRNA splicing and 3'-UTR truncation in round spermatids. Nucleic acids research 40**:** 7162-7175

Bienroth S, Wahle E, Suter-Crazzolara C, Keller W (1991) Purification of the cleavage and polyadenylation factor involved in the 3'-processing of messenger RNA precursors. The Journal of biological chemistry 266**:** 19768-19776

Blanco P, Sargent CA, Boucher CA, Howell G, Ross M, Affara NA (2001) A novel poly(A)-binding protein gene (PABPC5) maps to an X-specific subinterval in the Xq21.3/Yp11.2 homology block of the human sex chromosomes. Genomics 74**:** 1-11

Blechingberg J, Lykke-Andersen S, Jensen TH, Jorgensen AL, Nielsen AL (2007) Regulatory mechanisms for 3'-end alternative splicing and polyadenylation of the Glial Fibrillary Acidic Protein, GFAP, transcript. Nucleic acids research 35**:** 7636-7650

Boelens WC, Jansen EJ, van Venrooij WJ, Stripecke R, Mattaj IW, Gunderson SI (1993) The human U1 snRNP-specific U1A protein inhibits polyadenylation of its own pre-mRNA. Cell 72**:** 881-892

Brown KM, Gilmartin GM (2003) A mechanism for the regulation of pre-mRNA 3' processing by human cleavage factor Im. Molecular cell 12**:** 1467-1476

Castelo-Branco P, Furger A, Wollerton M, Smith C, Moreira A, Proudfoot N (2004) Polypyrimidine tract binding protein modulates efficiency of polyadenylation. Molecular and cellular biology 24**:** 4174-4183

Cevher MA, Zhang X, Fernandez S, Kim S, Baquero J, Nilsson P, Lee S, Virtanen A, Kleiman FE (2010) Nuclear deadenylation/polyadenylation factors regulate 3' processing in response to DNA damage. The EMBO journal 29**:** 1674-1687

Chennathukuzhi VM, Lefrancois S, Morales CR, Syed V, Hecht NB (2001) Elevated levels of the polyadenylation factor CstF 64 enhance formation of the 1kB Testis brain RNA-binding protein (TB-RBP) mRNA in male germ cells. Molecular reproduction and development 58**:** 460-469

Chipumuro E, Henriksen MA (2012) The ubiquitin hydrolase USP22 contributes to 3'-end processing of JAK-STAT-inducible genes. FASEB journal : official publication of the Federation of American Societies for Experimental Biology 26**:** 842-854

Chujo T, Ohira T, Sakaguchi Y, Goshima N, Nomura N, Nagao A, Suzuki T (2012) LRPPRC/SLIRP suppresses PNPase-mediated mRNA decay and promotes polyadenylation in human mitochondria. Nucleic acids research 40**:** 8033-8047

Chuvpilo S, Zimmer M, Kerstan A, Glockner J, Avots A, Escher C, Fischer C, Inashkina I, Jankevics E, Berberich-Siebelt F et al (1999) Alternative polyadenylation events contribute to the induction of NF-ATc in effector T cells. Immunity 10**:** 261-269

Coseno M, Martin G, Berger C, Gilmartin G, Keller W, Doublie S (2008) Crystal structure of the 25 kDa subunit of human cleavage factor Im. Nucleic acids research 36**:** 3474-3483

Dai W, Zhang G, Makeyev EV (2012) RNA-binding protein HuR autoregulates its expression by promoting alternative polyadenylation site usage. Nucleic acids research 40**:** 787-800

Dalziel M, Nunes NM, Furger A (2007) Two G-rich regulatory elements located adjacent to and 440 nucleotides downstream of the core poly(A) site of the intronless melanocortin receptor 1 gene are critical for efficient 3' end processing. Molecular and cellular biology 27**:** 1568-1580

Danckwardt S, Gantzert AS, Macher-Goeppinger S, Probst HC, Gentzel M, Wilm M, Grone HJ, Schirmacher P, Hentze MW, Kulozik AE (2011) p38 MAPK controls prothrombin expression by regulated RNA 3' end processing. Molecular cell 41**:** 298-310

Danckwardt S, Kaufmann I, Gentzel M, Foerstner KU, Gantzert AS, Gehring NH, Neu-Yilik G, Bork P, Keller W, Wilm M et al (2007) Splicing factors stimulate polyadenylation via USEs at non-canonical 3' end formation signals. The EMBO journal 26: 2658-2669

Dantonel JC, Murthy KG, Manley JL, Tora L (1997) Transcription factor TFIID recruits factor CPSF for formation of 3' end of mRNA. Nature 389: 399-402

Dass B, Tardif S, Park JY, Tian B, Weitlauf HM, Hess RA, Carnes K, Griswold MD, Small CL, Macdonald CC (2007) Loss of polyadenylation protein tauCstF-64 causes spermatogenic defects and male infertility. Proceedings of the National Academy of Sciences of the United States of America 104: 20374-20379

de Klerk E, Venema A, Anvar SY, Goeman JJ, Hu O, Trollet C, Dickson G, den Dunnen JT, van der Maarel SM, Raz V et al (2012) Poly(A) binding protein nuclear 1 levels affect alternative polyadenylation. Nucleic acids research 40: 9089-9101

de Vries H, Ruegsegger U, Hubner W, Friedlein A, Langen H, Keller W (2000) Human pre-mRNA cleavage factor II(m) contains homologs of yeast proteins and bridges two other cleavage factors. The EMBO journal 19: 5895-5904

Dettwiler S, Aringhieri C, Cardinale S, Keller W, Barabino SM (2004) Distinct sequence motifs within the 68-kDa subunit of cleavage factor Im mediate RNA binding, protein-protein interactions, and subcellular localization. The Journal of biological chemistry 279: 35788-35797

Di Giammartino DC, Shi Y, Manley JL (2013) PARP1 represses PAP and inhibits polyadenylation during heat shock. Molecular cell 49: 7-17

Edwards RA, Lee MS, Tsutakawa SE, Williams RS, Nazeer I, Kleiman FE, Tainer JA, Glover JN (2008) The BARD1 C-terminal domain structure and interactions with polyadenylation factor CstF-50. Biochemistry 47: 11446-11456

Elkon R, Drost J, van Haaften G, Jenal M, Schrier M, Vrielink JA, Agami R (2012) E2F mediates enhanced alternative polyadenylation in proliferation. Genome biology 13: R59

Feral C, Guellaen G, Pawlak A (2001) Human testis expresses a specific poly(A)-binding protein. Nucleic acids research 29: 1872-1883

Grange T, de Sa CM, Oddos J, Pictet R (1987) Human mRNA polyadenylate binding protein: evolutionary conservation of a nucleic acid binding motif. Nucleic acids research 15: 4771-4787

Gromak N, Rideau A, Southby J, Scadden AD, Gooding C, Huttelmaier S, Singer RH, Smith CW (2003) The PTB interacting protein raver1 regulates alpha-tropomyosin alternative splicing. The EMBO journal 22: 6356-6364

Gruber AR, Martin G, Keller W, Zavolan M (2012) Cleavage factor Im is a key regulator of 3' UTR length. RNA biology 9: 1405-1412

Gunderson SI, Polycarpou-Schwarz M, Mattaj IW (1998) U1 snRNP inhibits pre-mRNA polyadenylation through a direct interaction between U1 70K and poly(A) polymerase. Molecular cell 1**:** 255-264

Haddad R, Maurice F, Viphakone N, Voisinet-Hakil F, Fribourg S, Minvielle-Sebastia L (2012) An essential role for Clp1 in assembly of polyadenylation complex CF IA and Pol II transcription termination. Nucleic acids research 40**:** 1226-1239

Hall-Pogar T, Liang S, Hague LK, Lutz CS (2007) Specific trans-acting proteins interact with auxiliary RNA polyadenylation elements in the COX-2 3'-UTR. RNA 13**:** 1103-1115

Hirose Y, Manley JL (1998) RNA polymerase II is an essential mRNA polyadenylation factor. Nature 395**:** 93-96

Hockert KJ, Martincic K, Mendis-Handagama SM, Borghesi LA, Milcarek C, Dass B, MacDonald CC (2011) Spermatogenetic but not immunological defects in mice lacking the tauCstF-64 polyadenylation protein. Journal of reproductive immunology 89**:** 26-37

Huang Y, Li W, Yao X, Lin QJ, Yin JW, Liang Y, Heiner M, Tian B, Hui J, Wang G (2012) Mediator complex regulates alternative mRNA processing via the MED23 subunit. Molecular cell 45**:** 459-469

Hung LH, Heiner M, Hui J, Schreiner S, Benes V, Bindereif A (2008) Diverse roles of hnRNP L in mammalian mRNA processing: a combined microarray and RNAi analysis. RNA 14**:** 284-296

Jenal M, Elkon R, Loayza-Puch F, van Haaften G, Kuhn U, Menzies FM, Oude Vrielink JA, Bos AJ, Drost J, Rooijers K et al (2012) The poly(A)-binding protein nuclear 1 suppresses alternative cleavage and polyadenylation sites. Cell 149**:** 538-553

Jenny A, Hauri HP, Keller W (1994) Characterization of cleavage and polyadenylation specificity factor and cloning of its 100-kilodalton subunit. Molecular and cellular biology 14**:** 8183-8190

Ji X, Wan J, Vishnu M, Xing Y, Liebhaber SA (2013) alphaCP Poly(C) Binding Proteins Act as Global Regulators of Alternative Polyadenylation. Molecular and cellular biology 33**:** 2560-2573

Kaida D, Berg MG, Younis I, Kasim M, Singh LN, Wan L, Dreyfuss G (2010) U1 snRNP protects pre-mRNAs from premature cleavage and polyadenylation. Nature 468**:** 664-668

Kaneko S, Rozenblatt-Rosen O, Meyerson M, Manley JL (2007) The multifunctional protein p54nrb/PSF recruits the exonuclease XRN2 to facilitate pre-mRNA 3' processing and transcription termination. Genes & development 21**:** 1779-1789

Kashiwabara S, Zhuang T, Yamagata K, Noguchi J, Fukamizu A, Baba T (2000) Identification of a novel isoform of poly(A) polymerase, TPAP, specifically present in the cytoplasm of spermatogenic cells. Developmental biology 228**:** 106-115

Katahira J, Okuzaki D, Inoue H, Yoneda Y, Maehara K, Ohkawa Y (2013) Human TREX component Thoc5 affects alternative polyadenylation site choice by recruiting mammalian cleavage factor I. Nucleic acids research

Kaufmann I, Martin G, Friedlein A, Langen H, Keller W (2004) Human Fip1 is a subunit of CPSF that binds to U-rich RNA elements and stimulates poly(A) polymerase. The EMBO journal 23: 616-626

Keller W, Bienroth S, Lang KM, Christofori G (1991) Cleavage and polyadenylation factor CPF specifically interacts with the pre-mRNA 3' processing signal AAUAAA. The EMBO journal 10: 4241-4249

Kiefer H, Mizutani A, Iemura S, Natsume T, Ando H, Kuroda Y, Mikoshiba K (2009) Inositol 1,4,5-triphosphate receptor-binding protein released with inositol 1,4,5-triphosphate (IRBIT) associates with components of the mRNA 3' processing machinery in a phosphorylation-dependent manner and inhibits polyadenylation. The Journal of biological chemistry 284: 10694-10705

Kim H, Lee JH, Lee Y (2003) Regulation of poly(A) polymerase by 14-3-3epsilon. The EMBO journal 22: 5208-5219

Kim HS, Li H, Cevher M, Parmelee A, Fonseca D, Kleiman FE, Lee SB (2006) DNA damage-induced BARD1 phosphorylation is critical for the inhibition of messenger RNA processing by BRCA1/BARD1 complex. Cancer research 66: 4561-4565

Kleiman FE, Wu-Baer F, Fonseca D, Kaneko S, Baer R, Manley JL (2005) BRCA1/BARD1 inhibition of mRNA 3' processing involves targeted degradation of RNA polymerase II. Genes & development 19: 1227-1237

Ko B, Gunderson SI (2002) Identification of new poly(A) polymerase-inhibitory proteins capable of regulating pre-mRNA polyadenylation. Journal of molecular biology 318: 1189-1206

Kubo T, Wada T, Yamaguchi Y, Shimizu A, Handa H (2006) Knock-down of 25 kDa subunit of cleavage factor Im in Hela cells alters alternative polyadenylation within 3'-UTRs. Nucleic acids research 34: 6264-6271

Kyburz A, Friedlein A, Langen H, Keller W (2006) Direct interactions between subunits of CPSF and the U2 snRNP contribute to the coupling of pre-mRNA 3' end processing and splicing. Molecular cell 23: 195-205

Kyriakopoulou CB, Nordvarg H, Virtanen A (2001) A novel nuclear human poly(A) polymerase (PAP), PAP gamma. The Journal of biological chemistry 276: 33504-33511

Li W, Yeh HJ, Shankarling GS, Ji Z, Tian B, MacDonald CC (2012) The tauCstF-64 polyadenylation protein controls genome expression in testis. PloS one 7: e48373

Liang S, Lutz CS (2006) p54nrb is a component of the snRNP-free U1A (SF-A) complex that promotes pre-mRNA cleavage during polyadenylation. RNA 12: 111-121

Licatalosi DD, Mele A, Fak JJ, Ule J, Kayikci M, Chi SW, Clark TA, Schweitzer AC, Blume JE, Wang X et al (2008) HITS-CLIP yields genome-wide insights into brain alternative RNA processing. Nature 456**:** 464-469

Liu Y, Hu W, Murakawa Y, Yin J, Wang G, Landthaler M, Yan J (2013) Cold-induced RNA-binding proteins regulate circadian gene expression by controlling alternative polyadenylation. Scientific reports 3**:** 2054

Lou H, Helfman DM, Gagel RF, Berget SM (1999) Polypyrimidine tract-binding protein positively regulates inclusion of an alternative 3'-terminal exon. Molecular and cellular biology 19**:** 78-85

Lou H, Neugebauer KM, Gagel RF, Berget SM (1998) Regulation of alternative polyadenylation by U1 snRNPs and SRp20. Molecular and cellular biology 18**:** 4977-4985

Lutz CS, Alwine JC (1994) Direct interaction of the U1 snRNP-A protein with the upstream efficiency element of the SV40 late polyadenylation signal. Genes & development 8**:** 576-586

Lutz CS, Murthy KG, Schek N, O'Connor JP, Manley JL, Alwine JC (1996) Interaction between the U1 snRNP-A protein and the 160-kD subunit of cleavage-polyadenylation specificity factor increases polyadenylation efficiency in vitro. Genes & development 10**:** 325-337

Mandel CR, Kaneko S, Zhang H, Gebauer D, Vethantham V, Manley JL, Tong L (2006) Polyadenylation factor CPSF-73 is the pre-mRNA 3'-end-processing endonuclease. Nature 444**:** 953-956

Mansfield KD, Keene JD (2012) Neuron-specific ELAV/Hu proteins suppress HuR mRNA during neuronal differentiation by alternative polyadenylation. Nucleic acids research 40**:** 2734-2746

Martin G, Gruber AR, Keller W, Zavolan M (2012) Genome-wide analysis of pre-mRNA 3' end processing reveals a decisive role of human cleavage factor I in the regulation of 3' UTR length. Cell reports 1**:** 753-763

Martincic K, Alkan SA, Cheatle A, Borghesi L, Milcarek C (2009) Transcription elongation factor ELL2 directs immunoglobulin secretion in plasma cells by stimulating altered RNA processing. Nature immunology 10**:** 1102-1109

McCracken S, Fong N, Yankulov K, Ballantyne S, Pan G, Greenblatt J, Patterson SD, Wickens M, Bentley DL (1997) The C-terminal domain of RNA polymerase II couples mRNA processing to transcription. Nature 385**:** 357-361

McCracken S, Longman D, Johnstone IL, Caceres JF, Blencowe BJ (2003) An evolutionarily conserved role for SRm160 in 3'-end processing that functions independently of exon junction complex formation. The Journal of biological chemistry 278**:** 44153-44160

Mellman DL, Gonzales ML, Song C, Barlow CA, Wang P, Kendziorski C, Anderson RA (2008) A PtdIns4,5P2-regulated nuclear poly(A) polymerase controls expression of select mRNAs. Nature 451**:** 1013-1017

Millevoi S, Decorsiere A, Loulergue C, Iacovoni J, Bernat S, Antoniou M, Vagner S (2009) A physical and functional link between splicing factors promotes pre-mRNA 3' end processing. Nucleic acids research 37: 4672-4683

Millevoi S, Loulergue C, Dettwiler S, Karaa SZ, Keller W, Antoniou M, Vagner S (2006) An interaction between U2AF 65 and CF I(m) links the splicing and 3' end processing machineries. The EMBO journal 25: 4854-4864

Moreira A, Takagaki Y, Brackenridge S, Wollerton M, Manley JL, Proudfoot NJ (1998) The upstream sequence element of the C2 complement poly(A) signal activates mRNA 3' end formation by two distinct mechanisms. Genes & development 12: 2522-2534

Murthy KG, Manley JL (1995) The 160-kD subunit of human cleavage-polyadenylation specificity factor coordinates pre-mRNA 3'-end formation. Genes & development 9: 2672-2683

Nazeer FI, Devany E, Mohammed S, Fonseca D, Akukwe B, Taveras C, Kleiman FE (2011) p53 inhibits mRNA 3' processing through its interaction with the CstF/BARD1 complex. Oncogene 30: 3073-3083

Phillips C, Pachikara N, Gunderson SI (2004) U1A inhibits cleavage at the immunoglobulin M heavy-chain secretory poly(A) site by binding between the two downstream GU-rich regions. Molecular and cellular biology 24: 6162-6171

Pinto PA, Henriques T, Freitas MO, Martins T, Domingues RG, Wyrzykowska PS, Coelho PA, Carmo AM, Sunkel CE, Proudfoot NJ et al (2011) RNA polymerase II kinetics in polo polyadenylation signal selection. The EMBO journal 30: 2431-2444

Rosonina E, Ip JY, Calarco JA, Bakowski MA, Emili A, McCracken S, Tucker P, Ingles CJ, Blencowe BJ (2005) Role for PSF in mediating transcriptional activator-dependent stimulation of pre-mRNA processing in vivo. Molecular and cellular biology 25: 6734-6746

Rozenblatt-Rosen O, Nagaike T, Francis JM, Kaneko S, Glatt KA, Hughes CM, LaFramboise T, Manley JL, Meyerson M (2009) The tumor suppressor Cdc73 functionally associates with CPSF and CstF 3' mRNA processing factors. Proceedings of the National Academy of Sciences of the United States of America 106: 755-760

Ruegsegger U, Beyer K, Keller W (1996) Purification and characterization of human cleavage factor Im involved in the 3' end processing of messenger RNA precursors. The Journal of biological chemistry 271: 6107-6113

Ruegsegger U, Blank D, Keller W (1998) Human pre-mRNA cleavage factor Im is related to spliceosomal SR proteins and can be reconstituted in vitro from recombinant subunits. Molecular cell 1: 243-253

Ruzzenente B, Metodiev MD, Wredenberg A, Bratic A, Park CB, Camara Y, Milenkovic D, Zickermann V, Wibom R, Hultenby K et al (2012) LRPPRC is necessary for polyadenylation and coordination of translation of mitochondrial mRNAs. The EMBO journal 31: 443-456

Sartini BL, Wang H, Wang W, Millette CF, Kilpatrick DL (2008) Pre-messenger RNA cleavage factor I (CFIm): potential role in alternative polyadenylation during spermatogenesis. Biology of reproduction 78**:** 472-482

Shell SA, Hesse C, Morris SM, Jr., Milcarek C (2005) Elevated levels of the 64-kDa cleavage stimulatory factor (CstF-64) in lipopolysaccharide-stimulated macrophages influence gene expression and induce alternative poly(A) site selection. The Journal of biological chemistry 280**:** 39950-39961

Shi Y, Di Giammartino DC, Taylor D, Sarkeshik A, Rice WJ, Yates JR, 3rd, Frank J, Manley JL (2009) Molecular architecture of the human pre-mRNA 3' processing complex. Molecular cell 33**:** 365-376

Takagaki Y, Manley JL (2000) Complex protein interactions within the human polyadenylation machinery identify a novel component. Molecular and cellular biology 20**:** 1515-1525

Takagaki Y, Manley JL, MacDonald CC, Wilusz J, Shenk T (1990) A multisubunit factor, CstF, is required for polyadenylation of mammalian pre-mRNAs. Genes & development 4**:** 2112-2120

Takagaki Y, Ryner LC, Manley JL (1988) Separation and characterization of a poly(A) polymerase and a cleavage/specificity factor required for pre-mRNA polyadenylation. Cell 52**:** 731-742

Takagaki Y, Seipelt RL, Peterson ML, Manley JL (1996) The polyadenylation factor CstF-64 regulates alternative processing of IgM heavy chain pre-mRNA during B cell differentiation. Cell 87**:** 941-952

Topalian SL, Kaneko S, Gonzales MI, Bond GL, Ward Y, Manley JL (2001) Identification and functional characterization of neo-poly(A) polymerase, an RNA processing enzyme overexpressed in human tumors. Molecular and cellular biology 21**:** 5614-5623

Vagner S, Ruegsegger U, Gunderson SI, Keller W, Mattaj IW (2000a) Position-dependent inhibition of the cleavage step of pre-mRNA 3'-end processing by U1 snRNP. RNA 6**:** 178-188

Vagner S, Vagner C, Mattaj IW (2000b) The carboxyl terminus of vertebrate poly(A) polymerase interacts with U2AF 65 to couple 3'-end processing and splicing. Genes & development 14**:** 403-413

Venkataraman K, Brown KM, Gilmartin GM (2005) Analysis of a noncanonical poly(A) site reveals a tripartite mechanism for vertebrate poly(A) site recognition. Genes & development 19**:** 1315-1327

Veraldi KL, Arhin GK, Martincic K, Chung-Ganster LH, Wilusz J, Milcarek C (2001) hnRNP F influences binding of a 64-kilodalton subunit of cleavage stimulation factor to mRNA precursors in mouse B cells. Molecular and cellular biology 21**:** 1228-1238

Wahle E (1991) A novel poly(A)-binding protein acts as a specificity factor in the second phase of messenger RNA polyadenylation. Cell 66**:** 759-768

Wallace AM, Dass B, Ravnik SE, Tonk V, Jenkins NA, Gilbert DJ, Copeland NG, MacDonald CC (1999) Two distinct forms of the 64,000 Mr protein of the cleavage stimulation factor are expressed in mouse male germ cells. Proceedings of the National Academy of Sciences of the United States of America 96**:** 6763-6768

Xing H, Mayhew CN, Cullen KE, Park-Sarge OK, Sarge KD (2004) HSF1 modulation of Hsp70 mRNA polyadenylation via interaction with symplekin. The Journal of biological chemistry 279**:** 10551-10555

Yang H, Duckett CS, Lindsten T (1995) iPABP, an inducible poly(A)-binding protein detected in activated human T cells. Molecular and cellular biology 15**:** 6770-6776

Zhu ZH, Yu YP, Shi YK, Nelson JB, Luo JH (2009) CSR1 induces cell death through inactivation of CPSF3. Oncogene 28**:** 41-51
